# GRK Inhibition Potentiates Glucagon-Like Peptide-1 Action

**DOI:** 10.3389/fendo.2021.652628

**Published:** 2021-05-14

**Authors:** Seunghun P. Lee, Jenson Qi, Guozhang Xu, Matthew M. Rankin, James Littrell, June Zhi Xu, Ivona Bakaj, Alessandro Pocai

**Affiliations:** ^1^ Cardiovascular and Metabolic Disease Research, Janssen Research & Development, Spring House, PA, United States; ^2^ Discovery Sciences, Janssen Research & Development, Spring House, PA, United States

**Keywords:** GRK2 = G protein–coupled receptor kinase 2, GLP-1, diabetes, obesity, NASH, insulin secretion, CKD - chronic kidney disease

## Abstract

The glucagon-like peptide-1 receptor (GLP-1R) is a G-protein-coupled receptor (GPCR) whose activation results in suppression of food intake and improvement of glucose metabolism. Several receptor interacting proteins regulate the signaling of GLP-1R such as G protein-coupled receptor kinases (GRK) and β-arrestins. Here we evaluated the physiological and pharmacological impact of GRK inhibition on GLP-1R activity leveraging small molecule inhibitors of GRK2 and GRK3. We demonstrated that inhibition of GRK: i) inhibited GLP-1-mediated β-arrestin recruitment, ii) enhanced GLP-1-induced insulin secretion in isolated islets and iii) has additive effect with dipeptidyl peptidase 4 in mediating suppression of glucose excursion in mice. These findings highlight the importance of GRK to modulate GLP-1R function *in vitro* and *in vivo*. GRK inhibition is a potential therapeutic approach to enhance endogenous and pharmacologically stimulated GLP-1R signaling.

## Introduction

Glucagon-like peptide 1 receptor agonists (GLP-1 RAs) are an important therapy for patients with type 2 diabetes (T2D) given their ability to improve glucose metabolism and their associated weight loss, low risk for hypoglycemia and positive effects on cardiovascular outcomes (1, 2). Recent data also highlights the potential of this pathway in non-alcoholic steatohepatitis (3, 4), diabetic kidney disease (5, 6) and Alzheimer’s disease (7, 8). As a result, strategies that activate GLP-1R or stabilize active GLP-1 with pharmacological agonists or with Dipeptidyl Peptidase-4 Inhibitors (DPP-4i), are the subject of an intensive drug discovery effort (1, 9–11).

Another potential approach to potentiate and prolongate GLP-1R activation is by inhibiting proteins involved in termination of the receptor signaling. GLP-1R belongs to the Class B G-protein-coupled receptors (GPCRs) and GLP-1R stimulation leads to cAMP production, Ca^2+^ mobilization, and phosphorylation of ERK1/2 (pERK1/2) (1, 9, 12–14). For most GPCRs, homologous desensitization is thought to involve phosphorylation by G protein-coupled receptor kinases that results in recruitment of β-arrestins (15). In recombinant systems, GRK2 and β-arrestin has been reported to interact with GLP-1R in response to stimulation by GLP-1 (16, 17) and recently Arcones et al. demonstrated that GRK2 modulates GLP-1R (18). In this report, we leverage small molecule dual GRK2/3 (GRK) inhibitors (19) to evaluate whether pharmacological inhibition of G protein-coupled receptor kinases potentiates the physiological and pharmacological actions of GLP-1.

## Materials and Methods

### Reagents and Cell Lines

Human GLP-1 (7-36) amide used for activation of GLP-1R was obtained from Bachem (Torrance, CA). Fatty acid-free BSA was from SIGMA (St. Louis, MO). HBSS with calcium and magnesium without phenol red was purchased from Mediatech (Manassas, VA). The GRK2/3 (GRK compounds A and B) small molecule inhibitors used [compounds 8g and 8h described i (19)] were synthesized at Janssen Research & Development, LLC. PathHunter^®^ eXpress GLP-1R CHO-K1 β-Arrestin cells were obtained from Eurofins DiscoverX Corporation (Fremont, CA). F-12 and RPMI 1640 medium were from Gibco, a division of Thermo Fisher Scientific.

### GLP-1 Mediated β-Arrestin Recruitment Assay

PathHunter^®^ eXpress GLP1R CHO-K1 β-Arrestin cells were plated at 6000 cells/well in a 384-well, PDL, white and opaque plate in F12 medium containing 10% FBS, 0.3 mg/ml hygromycin, and 0.8 mg/ml G418. The plate was incubated for two days in a humidified incubator at 37°C and 5% CO_2_ prior to the experiment. On the day of the exeperiment, the cells were washed once with the Assay Buffer (HBSS with calcium and magnesium, 20 mM Hepes, and 0.1% fatty-acid free BSA) followed by a 10 minute preincubation with GRK inhibitors or vehicle (DMSO) at the indicated concentrations. The final DMSO concentration was 0.1%. GLP-1 was added to the indicated concentrations, and incubation was continued for an additional 90 minutes at 37°C. The detection reagent was then added the cells, followed by a 60 minute incubation at the room temperature. The plate was read on MicroBeta LumiJet (PerkinElmer, Waltham, MA). In this cellular system, the GLP-1 receptor is fused in frame with the small enzyme fragment of ProLinkTM and co-expressed in cells expressing a fusion of the larger deletion mutant of β-gal, also called enzyme acceptor. Activation of GLP-1 receptor stimulates the binding of β-arrestin to the ProLinkTM tagged GLP-1 receptor and forces complementation of the two enzyme fragments, resulting in the formation of an active β-gal enzyme. The cellular β-gal enzyme activity is measured using chemiluminescent detection reagents.

### GLP-1 Mediated Insulin Secretion in INS-1 832/13 Cells

INS-1 832/13 cells were cultured in medium composed of RPMI 1640 medium supplemented with 10% fetal bovine serum, 50 IU/mL penicillin, 50 mg/L streptomycin, 10 mM HEPES, 2 mM L-glutamine, 1 mM sodium pyruvate, and 50 µM beta-mercaptoethanol. Cells were split twice a week, grown in a 37°C incubator under a humidified atmosphere containing 5% CO_2_ and plated at (60,000 cells/well) in a 96-well plate, two days prior to the experiment. Cells were washed twice with glucose-free Krebs-Ringer Bicarbonate buffer (KRB) (116 mM NaCl, 1.8 mM CaCl_2_, 0.8 mM MgSO_4_, 5.4 mM KCl, 1 mM NaH_2_PO_4_, 26 mM NaHCO_3_, and 0.2% fatty-acid free BSA, pH 7.4) followed by preincubation for 1 h at 37°C in glucose-free KRB and the indicated concentration of GRK compounds or vehicle (DMSO) were added. The final DMSO concentration was 0.1%. After 30 min incubation at 37°C, 2 mM or 5 mM glucose and 10 nM GLP-1 were added to the wells for a final volume of 200 μL. After further incubation for 1 hour at 37°C, 20 μL of the culture supernatant was diluted 1:15 in the Dilution Buffer provided in the Insulin High Range Assay HTRF Kit (Cisbio, Bedford, MA). Insulin was detected using the Insulin High Range Assay HTRF Kit according to the manufacturer’s instructions (Cisbio, Bedford, MA). All results were obtained in 5 replicates.

### Glucose Stimulated Insulin Secretion (GSIS) in Isolated Mouse Pancreatic Islets

Islets were isolated from mouse pancreas by collagenase digestion (20). The collagenase was dissolved in Hanks’ balanced salt solution (Invitrogen, Carlsbad, CA) containing 10 g/ml DNase I (Roche Diagnostics, Indianapolis, IN) and 0.2% BSA, and was subsequently injected into the common bile duct. The pancreas was removed and incubated at 37°C for 20 min and washed three times with Hanks’ balanced salt solution and islets were collected under a microscope. Purified islets were used after an overnight incubation in RPMI 1640 medium containing 5.5 mmol/l glucose and 10% fetal bovine serum in a humidified atmosphere containing 5% CO_2_/95% air at 37°C. A group of 7 islets was preincubated in Krebs Ringer bicarbonate buffer (KRBH) containing 0.2% BSA and 2 mM glucose for 40 min at 37°C. The indicated concentration of GRK compounds or vehicle (DMSO) were added. The final DMSO concentration was 0.1%. After 30 min incubation at 37°C, 2 mM or 10 mM glucose along with 20 nM GLP-1 were added to wells. After further incubation for 1 hour at 37°C with shaking, culture supernatant was collected, and insulin was detected using the Insulin High Range Assay HTRF Kit according to the manufacturer’s instructions (Cisbio, Bedford, MA).

### Real-Time PCR Assay of mRNA Abundance of GRK1-6 in Mouse Pancreatic Islets

Total RNA was extracted from the isolated islets using Trizol (Thermo Fisher Scientific) and chloroform, followed by the steps according to the RNeasy Mini kit (Qiagen). The purity and concentration of RNA was determined by measuring the absorbance at 260 nm and 280 nm with the NanoDrop Spectrophotometer. cDNA was synthesized using the Superscript IV VILO Reverse Transcriptase Kit (Thermo Fisher Scientific). Quantitative real-time PCR data was generated using the ViiA7 Real-time PCR System (Thermo Fisher Scientific). Samples were run in duplicate. Cyclophilin A (PPIA) was used as a housekeeping gene, and quantification of the data was generated using the 2ΔΔCt method. The primers used were purchased from Thermo Fisher Scientific, and their catalog numbers were as follows: PPIA (Cat#Mm02342430_g1), Grk1 (Cat#Mm01220712_m1), Adrbk1 (Grk2, Cat#Mm00804778_m1), Adrbk2 (Grk3, Cat#Mm00622042_m1), Grk4 (Cat#Mm01213690_m1), Grk5 (Cat#Mm00517039_m1), Grk6 (Cat#00442425_m1).

### Oral Glucose Tolerance Test (OGTT) in C57BL/6J Mice

Three-month old male C57BL/6J mice with free access to water were fasted overnight. After baseline weight and glucose were collected, mice were randomized into groups with equal weight. GRK inhibitors along with DPP4i formulated in 20% 2-Hydroxypropyl)-β-cyclodextrin (HPBCD, Sigma-Aldrich) or vehicle 20% HPBCD were provided by oral administration 60 minutes prior to an oral glucose challenge. Mice received oral gavage of glucose (2 g/kg; 20% glucose, 10 ml/kg). Blood samples were collected at time 0 (just before glucose load), 15, 30, 60, and 120 minutes after glucose administration. Six mice per group and data point were used. Plasma was obtained by centrifugation and stored at -20C for further analysis. The Institutional Animal Care and Use Committee (IACUC) of Janssen Research & Development, LLC. approved all animal procedures.

### Biochemical Analysis

Insulin was measured by ELISA (Linco/Millipore). Free fatty acids (FFA) and ketone bodies were measured using commercially available enzyme-coupled spectrophotometric assays (Wako Chemicals, Richmond, VA). Blood glucose levels were measured using a OneTouch glucometer (LifeScan, Milpitas, CA).

### Statistical Analysis

All data are presented as means ± SE. Comparisons among groups were made using 1-way ANOVA, 2-way ANOVA or unpaired Student’s t-test using GraphPad Prism statistical software.

P < 0.05 was regarded as statistically significant.

## Results

### GRK Inhibition Reduces GLP-1 Mediated β-Arrestin Recruitment

**Figure 1:**
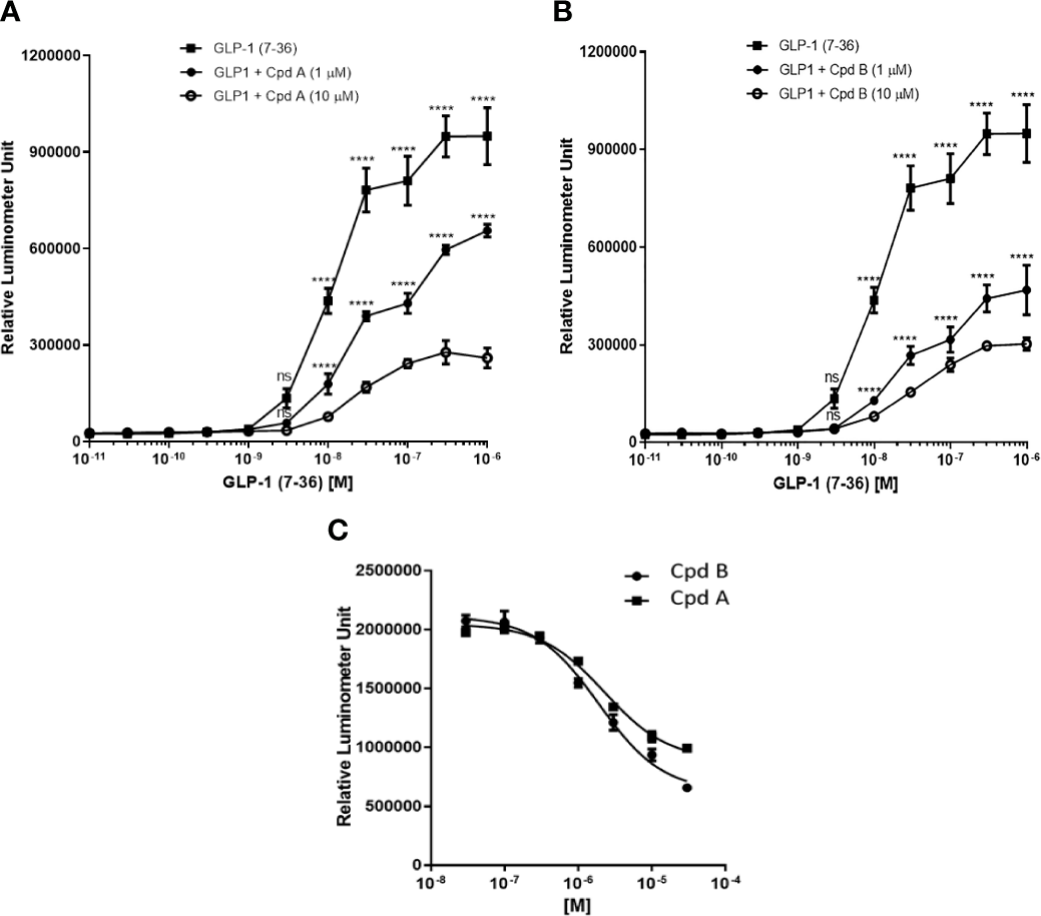
GLP-1-stimulated recruitment of β-arrestin in PathHunter^®^ eXpress GLP1R CHO-K1 β-arrestin cells. **(A)** Inhibition of GLP-1-stimulated recruitment of β-arrestin by Cpd A; **(B)** Inhibition of GLP-1-stimulated recruitment of β-arrestin by Cpd B; **(C)** IC_50_ determination of Cpd A and Cpd B Values represent the mean of three data points ± SEM. For **(A, B)** Statistical comparison are assessed by one-way ANOVA with Dunnett’s multiple comparisons test. ****P < 0.0001; ns, not significant.

To confirm that GLP-1 can induce the recruitment of GRK2 and β-arrestin to the GLP-1 receptor we used two small molecule GRK2/3 (GRK) inhibitors (Cpd A and B) with good selectivity and permeability (19). Both compounds displayed >300-fold selectivity against GRK1, GRK4, GRK5, GRK6, and GRK7 (19) and similar inhibition of GRK3, a member of the G protein-coupled receptor kinases highly homologous to GRK2 (**Table 1**). In CHO-K1 cell line stably expressing human GLP-1 receptor and β-arrestin, the addition of GLP1(7-36) led to a dose-dependent increase of cellular β-gal enzyme activity (**Figure 1A**). The EC_80_ value for human GLP1(7-36) was approximately 100 nM. Pre-incubation of Cpd A and B at 1 or 10 μM with CHO-K1/β-arrestin/GLP-1R cells reduced GLP1(7-36)-stimulated cellular β-gal enzyme activity (**Figures 1A, B**). The IC_50_ value for Cpd A and Cpd B to inhibit 100 nM human GLP1(7-36)-stimulated β-arrestin recruitment to the GLP-1R was 1.9 and 2.3 μM respectively (**Figure 1C**).

**Table 1:** Kinase activity profile of Compound A and B against GRK2, GRK1, GRK3, GRK4, GRK5, GRK6, GRK7, and other kinases.

Kinases	Compound A	Compound B
GRK2	19	10
GRK1	>10000	>10000
GRK3*	6	3
GRK4*	>3000	>3000
GRK5	>10000	4497
GRK6	>10000	3270
GRK7	>10000	9137
PKA	4880	855
PKBα	>10000	1449
PKCα	>10000	>10000
PKCβ1	>10000	9068
CaMKIIβ	>10000	3846
ROCK-1	1271	246
Aurora-A	137	11
Rsk1	3151	483

*Ki value

### GRK Inhibition Potentiates GLP-1 Mediated Insulin Secretion in INS-1 832/13 Cells

**Figure 2:**
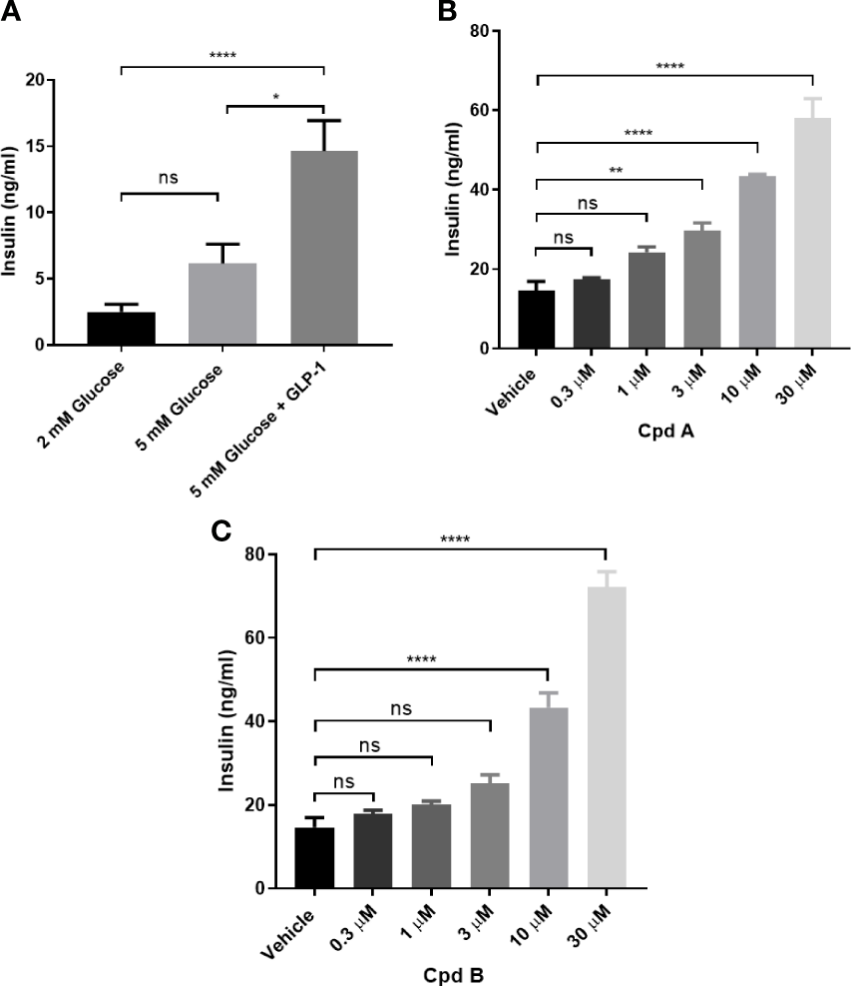
GLP-1 mediated insulin secretion in INS-1 832/13 cells**. (A)** GLP-1 mediated insulin secretion; **(B)** Effect of Cpd A on 10 nM GLP-1 mediated insulin secretion at 5 mM glucose; **(C)** Effect of Cpd B on 10 nM GLP-1 mediated insulin secretion at 5 mM glucose. Values represent the mean of five replicates ± SEM. Statistical comparison for more than 2 datasets are assessed by one-way ANOVA with Dunnett’s multiple comparisons test. *P = 0.01-0.1, **P = 0.001-0.01, ****P < 0.0001; ns, not significant. Unpaired t-test was performed for 2 datasets.

We then tested whether GRK is involved in GLP-1 mediated insulin secretion in INS-1 832/13 cells. GLP-1 at 10 nM stimulated insulin secretion in the presence of 5 mM glucose (**Figure 2A**) and pre-incubation with GRK inhibitors potentiated insulin secretion stimulated by GLP-1 while GRK inhibition alone had no effect (**Figures 2B, C**).

### GRK Inhibition Potentiates GLP-1 Mediated Glucose-Stimulated Insulin Secretion (GSIS) in Mouse Isolated Islets

**Figure 3:**
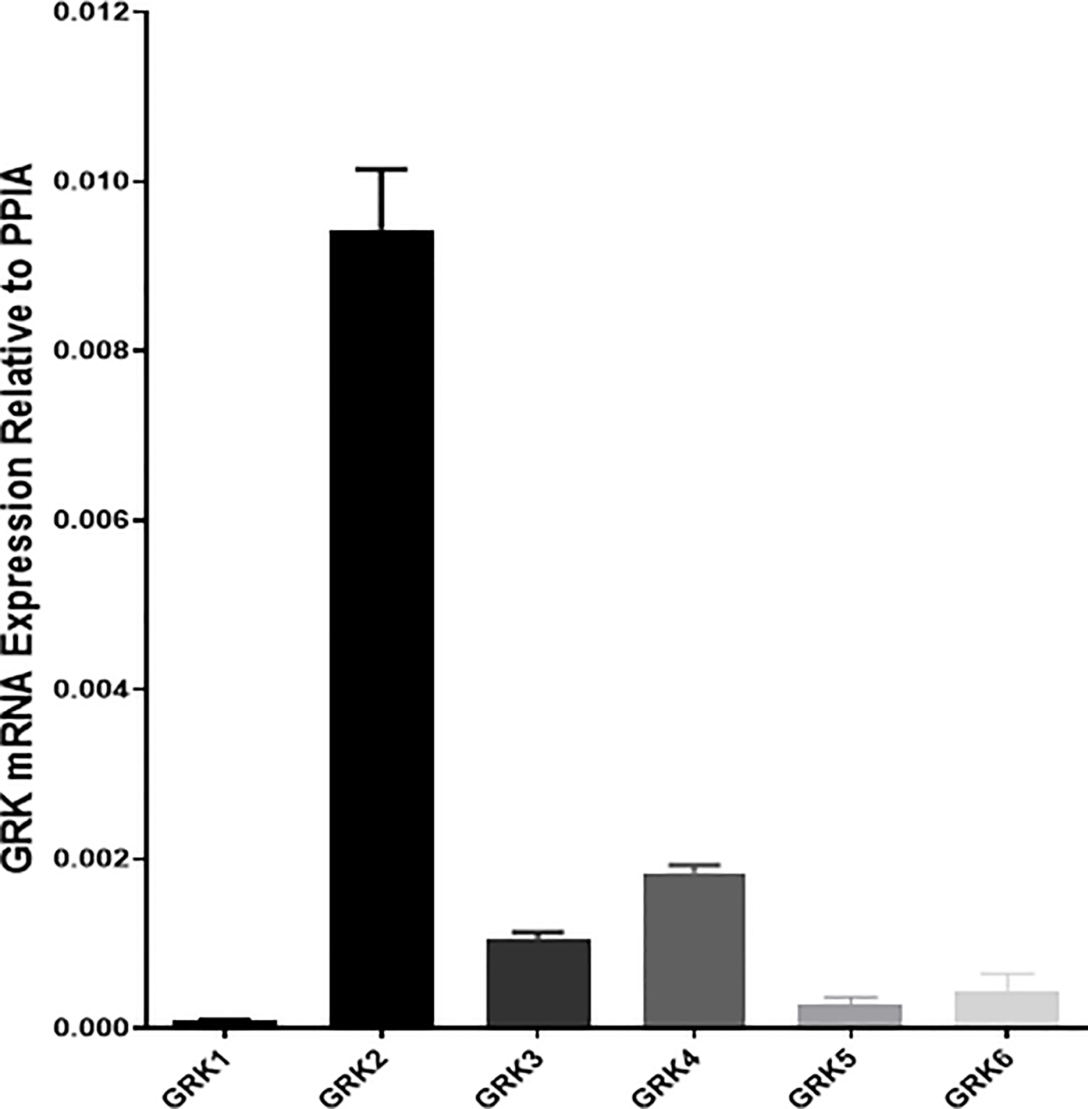
Real-time PCR assay of mRNA abundance of GRK1-6 in mouse pancreatic islets. Cyclophilin A (PPIA) was used as a housekeeping gene, and quantifications were conducted using the 2ΔΔCt method. Samples were run in duplicates.

**Figure 4:**
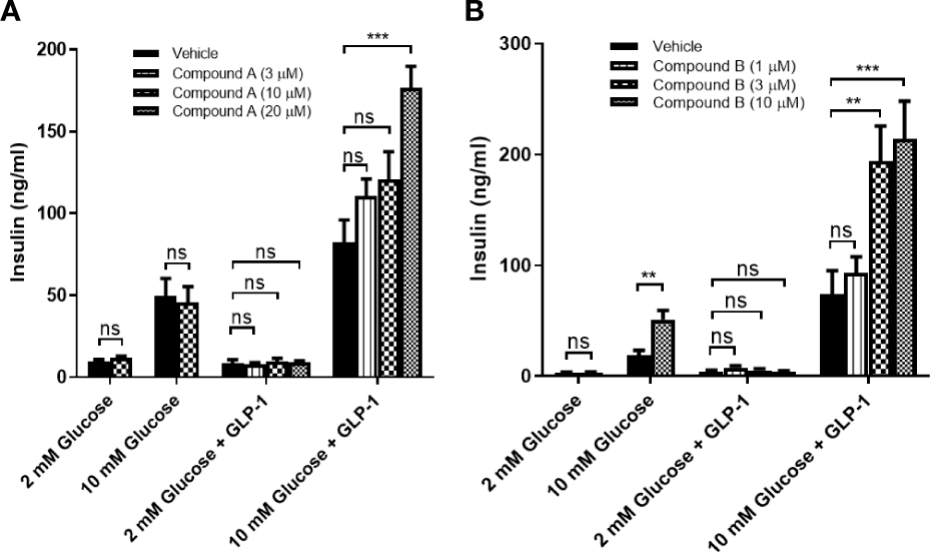
GLP-1 mediated insulin secretion in mouse pancreatic islets. **(A)** Effect of Cpd A on 20 nM GLP-1 mediated insulin secretion at 2 mM glucose or 10 mM glucose; **(B)** Effect of Cpd B on 20 nM GLP-1 mediated insulin secretion at 2 mM glucose or 10 mM glucose. Values represent the mean of eight replicates ± SEM. Statistical comparison for more than 2 datasets are assessed by one-way ANOVA with Dunnett’s multiple comparisons test. **P = 0.001–0.01, ***P < 0.001; ns, not significant. An unpaired t-test is performed for 2 datasets.

We first confirmed that all G protein receptor Kinases isoforms are expressed in mouse islets with GRK2 showing the highest mRNA expression (**Figure 3**) (21). Next, we investigated the effect of GRK inhibition on GLP-1 mediated GSIS in mouse isolated islets. GLP-1 and GLP-1+ Cpd A or Cpd B did not stimulate insulin secretion when incubated with 2 mM glucose (**Figures 4A, B**). At 10 mM glucose, Cpd A and Cpd B dose-dependently potentiated GLP-1 mediated GSIS (**Figures 4A, B**). Pre-incubation of Cpd A alone up to 20 μM did not affect GSIS while Cpd B resulted in a small but significant increase in GSIS (**Figures 4A, B**).

### GRK Inhibition and DPP-4i-Mediated Suppression of Glucose Excursion in Mice

**Figure 5:**
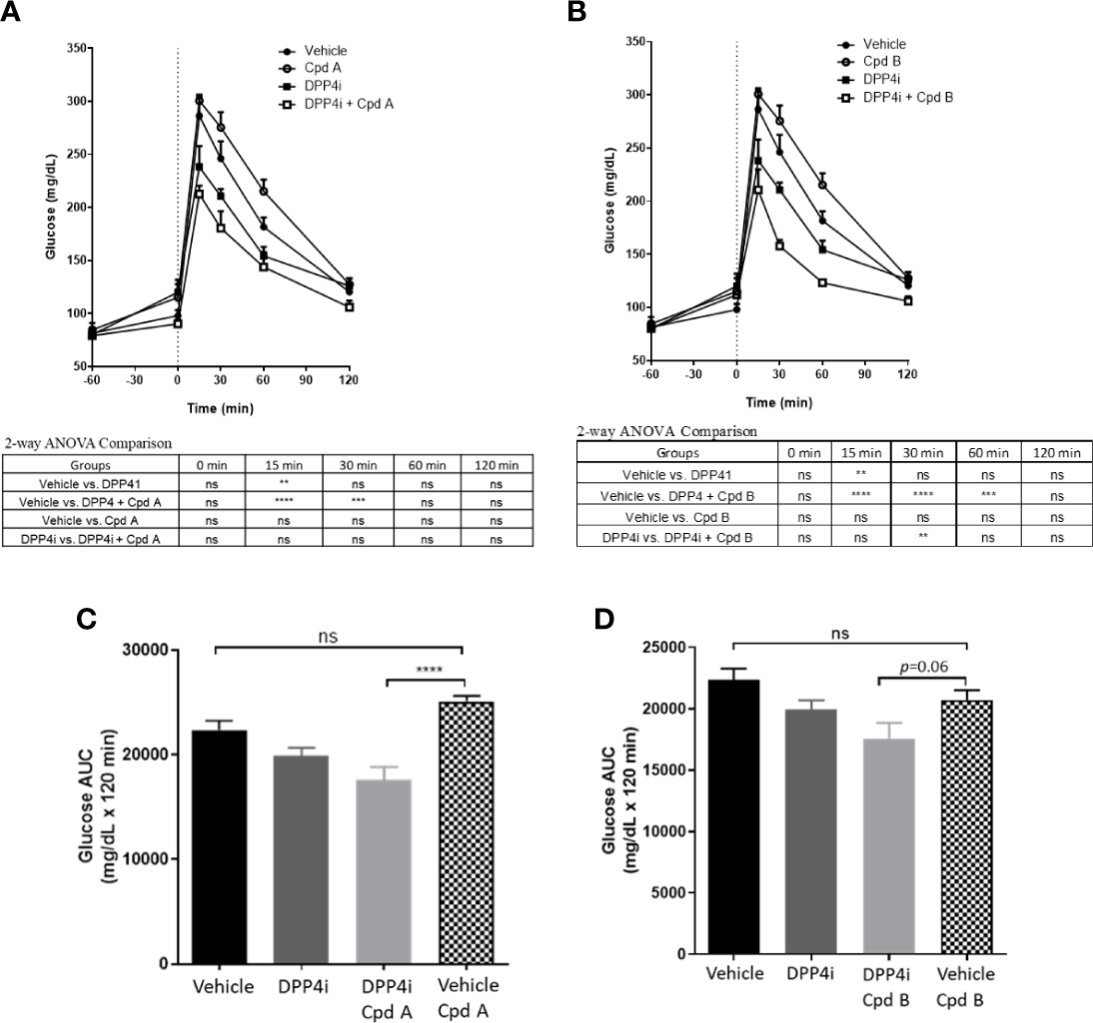
OGTT in C57BL/6J mice. **(A)** Effect of Cpd A on DPP4i-mediated glucose excursion in C57BL/6J mice; **(B)** Effect of Cpd B on DPP4i-mediated glucose excursion in C57BL/6J mice; **(C)** Results of A expressed as Area under the curve (AUC); **(D)** Results of A expressed as AUC. Data are expressed as mean values ± SD of 6 animals in each group. For Figures **(A, B)** differences between group means are assessed by Ordinary 2-way ANOVA followed by Tukey’s multiple comparison test. The analysis result was shown under each figure. For Figures **(C, D)**, differences between group means are assessed by Ordinary one-way ANOVA followed by Tukey’s multiple comparison test. **P = 0.001-0.01, ***P = 0.0001-0.001, ****P < 0.0001; ns, not significant.

Overnight fasted mice were administrated a GRK inhibitor, DPP4i, or DPP4i with a GRK inhibitor 60 min prior to a glucose challenge. In the presence of plasma concentrations required for GRK inhibition (**Table 2**), Cpd A and Cpd B alone had no effect on glucose excursion (**Figures 5A, B**). Cpd A administration with DPP4i resulted in a significant glucose lowering versus Cpd A at 15, 30 and 60 min after the glucose challenge (**Figure 5A**) resulting in significant reduction of the overall area under the curve (AUC) (**Figure 5C**). In a similar study, Cpd B administration with DPP-4i significantly lowered plasma glucose at 15, 30 and 60 min (**Figure 5D**), with a trend to reduce the overall glucose excursion (*p*=0.06) (**Figure 5D**).

**Table 2:** Plasma GRK2 inhibitors concentration at the end of the OGTT study.

Group	Cpd A	DPP4i + Cpd A	Cpd B	DPP4i + Cpd B
Plasma Concentration (μM)	4.25 ± 0.87	5.79 ± 0.64	4.82 ± 0.095	5.00 ± 0.080

Data expressed as mean (6 mice) ± S.D.

## Discussion

G protein receptor kinases is a family of seven serine/threonine protein kinases that specifically recognize and phosphorylate activated GPCRs. Receptor phosphorylation triggers the binding of β-arrestin potentially contributing to decrease the response of the receptor to the respective agonist. In addition to these phosphorylation-dependent processes, GRKs may also modulate cellular responses in a phosphorylation-independent manner due to their ability to interact with a variety of proteins involved in signaling and trafficking such as Gαq and Gs subunits, PI3K, clathrin, caveolin, MEK, and AKT (22, 23).

GLP-1R activation has been shown to lead to β-arrestin and GRK2 recruitment (16, 17). In the present report, we confirmed that GLP-1 dose-dependently stimulated the interaction between GLP-1R and β-arrestin (**Figure 1A**) and that pharmacological inhibition of GRK enhanced the effect of GLP-1 while the interaction between β-arrestin and GLP-1R is reduced. These data support the notion that GRK inhibition attenuate the recruitment of β-arrestin to GLP-1R *via* phosphorylation of the C-terminal of GLP-1R.

GLP-1-mediated activation of GLP-1R, leading to Gαs-mediated intracellular cAMP production, Ca^2+^ mobilization, and ERK1/2 phosphorylation has been shown to stimulate insulin secretion (12, 14). Leveraging small molecules recently discovered (19) we showed that inhibition of GRK2/3 results in potentiation of GLP-1 stimulated insulin secretion in INS832/13 and in mouse islets. Lastly, following stimulation of endogenous incretin secretion by an oral glucose tolerance test, we demonstrated for the first time that inhibition of GRK and DPP-4i resulted in additive suppression of glucose excursion in mice, while GRK2 inhibition alone had no effect. In our study the effect of DPP-4 on glucose tolerance was not significantly potentiated by GRK inhibition. Because GRK2 levels and activity have been reported to be enhanced in obesity and insulin resistance (24), in future studies it will be important to evaluate whether GRK inhibition enhances the effect of DPP-4. Recently, Arcones et al. demonstrated that reduced GRK2 levels potentiate insulin release in mice in response to the GLP-1R agonist Exendin-4 supporting the notion that GRK2 is an important negative modulator of GLP-1R action (18).

Given that several DPP4i and GLP-1RA have been approved or in clinical development for diabetes and other obesity-related metabolic complications (1, 3–6, 25), in future studies it will be important to confirm that selective pharmacological inhibition of GRK2 mediates the overall actions of GLP-1.

A potential role for GRK2/beta-arrestin-1 system in modulating GIP actions is still controversial (25–27). It is possible that in addition to GLP-1R activation, GIP-R may contribute to the additive glucose-lowering effect observed in our study with DPP-4i and GRK inhibition. Recently, Tirzepatide, a dual GIP/GLP-1 receptor agonist demonstrated superior glucose and body-weight lowering properties to GLP-1 receptor agonism (GLP-1RA) in T2D subjects and (28) so it will be important to assess whether GIP-R is involved in the effect of GRK inhibition. Future studies will need to consider potential biased agonism by different GLP-1 agonists which could differentially impact GRK-mediated β-arrestin recruitment (14, 16, 29–31) and result in differential pharmacological effects and evaluate the role of GRK inhibition in models with impaired glucose tolerance (24).

GRK inhibition is a new potential approach to potentiate the physiological and pharmacological actions of GLP-1 and can be leveraged alone or in fixed-dosed combinations with DPP4i or GLP1-RA.

## Data Availability Statement

The original contributions presented in the study are included in the article/supplementary material. Further inquiries can be directed to the corresponding author.

## Ethics Statement

The animal study was reviewed and approved by IACUC Janssen.

## Author Contributions

MR, IB, JQ, SPL, JL, JZX, and GX contributed to the experimental design and executed the studies. JQ, SPL, and AP designed the studies and wrote the manuscript. All authors contributed to the article and approved the submitted version.

## Conflict of Interest

The authors are employees of Janssen.

## References

[1] Müller TD Finan B Bloom SR D’Alessio D Drucker DJ Flatt PR. Glucagon-like Peptide 1 (GLP-1). Mol Metab (2019) 30:72–130. 10.1016/j.molmet.2019.09.010 31767182 PMC6812410

[2] Dalsgaard NB Brønden A Vilsbøll T Knop FK. Cardiovascular Safety and Benefits of GLP-1 Receptor Agonists. Expert Opin Drug Saf (2017) 16(3):351–63. 10.1080/14740338.2017.1281246 28102093

[3] Bifari F Manfrini R Dei Cas M Berra C Siano M Zuin M. Multiple Target Tissue Effects of GLP-1 Analogues on non-Alcoholic Fatty Liver Disease (NAFLD) and non-Alcoholic Steatohepatitis (NASH). Pharmacol Res (2018) 137:219–29. 10.1016/j.phrs.2018.09.025 30359962

[4] Newsome PN Buchholtz K Cusi K Linder M Okanoue T Ratziu V. Nn9931-4296 Investigators. A Placebo-Controlled Trial of Subcutaneous Semaglutide in Nonalcoholic Steatohepatitis. N Engl J Med (2020) 384(12):1113–24. 10.1056/NEJMoa2028395 33185364

[5] Giugliano D De Nicola L Maiorino MI Bellastella G Esposito K. Type 2 Diabetes and the Kidney: Insights From Cardiovascular Outcome Trials. Diabetes Obes Metab (2019) 21(8):1790–800. 10.1111/dom.13743 30969018

[6] Holst JJ. Incretin Therapy for Diabetes Mellitus Type 2. Curr Opin Endocrinol Diabetes Obes (2020) 27(1):2–10. 10.1097/MED.0000000000000516 31815785

[7] Irwin N Flatt PR. New Perspectives on Exploitation of Incretin Peptides for the Treatment of Diabetes and Related Disorders. World J Diabetes (2015) 6(15):1285–95. 10.4239/wjd.v6.i15.1285 PMC4635139 26557956

[8] Available at: https://www.globenewswire.com/news-release/2020/12/16/2146164/0/en/Novo-Nordisk-to-enter-phase-3-development-in-Alzheimer-s-disease-with-oral-semaglutide.html.

[9] Baggio LL Drucker DJ. Biology of Incretins: GLP-1 and GIP. Gastroenterology (2007) 132(6):2131–57. 10.1053/j.gastro.2007.03.054 17498508

[10] Pocai A. Unraveling Oxyntomodulin, GLP1’s Enigmatic Brother. J Endocrinol (2012) 215(3):335–46. 10.1530/JOE-12-0368 PMC3493657 23019069

[11] Pathak NM Pathak V Gault VA McClean S Irwin N Flatt PR. Novel Dual Incretin Agonist Peptide With Antidiabetic and Neuroprotective Potential. Biochem Pharmacol (2018) 155:264–74. 10.1016/j.bcp.2018.07.021 30028989

[12] Koole C Wootten D Simms J Valant C Sridhar R Woodman OL. Allosteric Ligands of the Glucagon-Like Peptide 1 Receptor (GLP-1R) Differentially Modulate Endogenous and Exogenous Peptide Responses in a Pathway-Selective Manner: Implications for Drug Screening. Mol Pharmacol (2010) 78(3):456–65. 10.1124/mol.110.065664 PMC3202488 20547734

[13] Couvineau A Laburthe,M. The Family B1 GPCR: Structural Aspects and Interaction With Accessory Proteins. Curr Drug Targets (2012) 13:103–15. 10.2174/138945012798868434 21777182

[14] Wootten D Reynolds CA Smith KJ Mobarec JC Koole C Savage EE. The Extracellular Surface of the GLP-1 Receptor Is a Molecular Trigger for Biased Agonism. Cell (2016) 165:1632–43. 10.1016/j.cell.2016.05.023 PMC4912689 27315480

[15] Widmann C Dolci W Thorens B. Internalization and Homologous Desensitization of the GLP-1 Receptor Depend on Phosphorylation of the Receptor Carboxyl Tail At the Same Three Sites. Mol Endocrinol (1997) 11(8):1094–102. 10.1210/mend.11.8.9959 9212057

[16] Jorgensen R Kubale V Vrecl M Schwartz TW Elling CE. Oxyntomodulin Differentially Affects Glucagon-Like Peptide-1 Receptor Beta-Arrestin Recruitment and Signaling Through Galpha(s). J Pharmacol Exp Ther (2007) 322(1):148–54. 10.1124/jpet.107.120006 17395766

[17] Jorgensen R Norklit Roed S Heding A Elling CE. Beta-Arrestin2 as a Competitor for GRK2 Interaction With the GLP-1 Receptor Upon Receptor Activation. Pharmacology (2011) 88:174–81. 10.1159/000330742 21952200

[18] Arcones AC Vila-Bedmar R Mirasierra M Cruces-Sande M Vallejo M Jones B. GRK2 Regulates GLP-1R-mediated Early Phase Insulin Secretion In Vivo. BMC Biol (2021) 19(1):40. 10.1186/s12915-021-00966-w 33658023 PMC7931601

[19] Xu G Gaul MD Liu Z DesJarlais RL Qi J Wang W. Hit-to-lead Optimization and Discovery of a Potent, and Orally Bioavailable G Protein Coupled Receptor Kinase 2 (GRK2) Inhibitor. Bioorg Med Chem Lett (2020) 30(23):127602. 10.1016/j.bmcl.2020.127602 33038544

[20] Sutton R Peters M McShane P Gray DW Morris PJ. Isolation of Rat Pancreatic Islets by Ductal Injection of Collagenase. Transplantation (1986) 42:689–91. 10.1097/00007890-198612000-00022 3024372

[21] Benovic JL Onorato JJ Arriza JL Stone WC Lohse M Jenkins NA. Cloning, Expression, and Chromosomal Localization of Beta-Adrenergic Receptor Kinase 2. A New Member of the Receptor Kinase Family. J Biol Chem (1991) 266:14939–46. 10.1016/S0021-9258(18)98568-6 1869533

[22] Kohout TA Lefkowitz RJ. Regulation of G Protein-Coupled Receptor Kinases and Arrestins During Receptor Desensitization. Mol Pharmacol (2003) 63:9–18. 10.1124/mol.63.1.9 12488531

[23] Penela P Ribas C Mayor J. Mechanisms of Regulation of the Expression and Function of G Protein-Coupled Receptor Kinases. Cell Signal (2003) 15:973–81. 10.1016/s0898-6568(03)00099-8 14499340

[24] Murga C Arcones AC Cruces-Sande M Briones AM Salaices M Mayor F. G Protein-Coupled Receptor Kinase 2 (GRK2) as a Potential Therapeutic Target in Cardiovascular and Metabolic Diseases. Front Pharmacol (2019) 19;10:112. 10.3389/fphar.2019.00112 PMC6390810 30837878

[25] Al-Sabah S Al-Fulaij M Shaaban G Ahmed HA Mann RJ Donnelly,D. The GIP Receptor Displays Higher Basal Activity Than the GLP-1 Receptor But Does Not Recruit GRK2 or Arrestin3 Effectively. PloS One (2014) 9:e106890. 10.1371/journal.pone.0106890 25191754 PMC4156404

[26] Tseng CC Zhang XY. Role of G Protein-Coupled Receptor Kinases in Glucose-Dependent Insulinotropic Polypeptide Receptor Signaling. Endocrinology (2000) 141(3):947–52. 10.1210/endo.141.3.7365 10698169

[27] Abdullah N Beg M Soares D Dittman JS McGraw TE. Downregulation of a GPCR by β-Arrestin2-Mediated Switch From an Endosomal to a TGN Recycling Pathway. Cell Rep (2016) 17(11):2966–78. 10.1016/j.celrep.2016.11.050 PMC5161243 27974210

[28] Available at: https://www.prnewswire.com/news-releases/tirzepatide-achieved-superior-a1c-and-body-weight-reductions-across-all-three-doses-compared-to-injectable-semaglutide-in-adults-with-type-2-diabetes-301239948.html.

[29] Darbalaei S Yuliantie E Dai A Chang R Zhao P Yang D. Evaluation of Biased Agonism Mediated by Dual Agonists of the GLP-1 and Glucagon Receptors. Biochem Pharmacol (2020) 180:114150. 10.1016/j.bcp.2020.114150 32682761

[30] Willard FS Douros JD Gabe MB Showalter AD Wainscott DB Suter TM. Tirzepatide is an Imbalanced and Biased Dual GIP and GLP-1 Receptor Agonist. JCI Insight (2020) 5(17):e140532. 10.1172/jci.insight.140532 PMC7526454 32730231

[31] Kim D Wang L Beconi M Eiermann GJ Fisher MH He H. (2r)-4-oxo-4-[3-(trifluoromethyl)-5,6-dihydro[1,2,4]triazolo[4,3-a]pyrazin-7(8H)-yl]-1-(2,4,5-trifluorophenyl)butan-2-amine: A Potent, Orally Active Dipeptidyl Peptidase IV Inhibitor for the Treatment of Type 2 Diabetes. J Med Chem (2005) 13;48(1):141–51. 10.1021/jm0493156 15634008

